# Comparative methylome analysis identifies new tumour subtypes and biomarkers for transformation of nephrogenic rests into Wilms tumour

**DOI:** 10.1186/s13073-015-0136-4

**Published:** 2015-02-02

**Authors:** Jocelyn Charlton, Richard D Williams, Neil J Sebire, Sergey Popov, Gordan Vujanic, Tasnim Chagtai, Marisa Alcaide-German, Tiffany Morris, Lee M Butcher, Paul Guilhamon, Stephan Beck, Kathy Pritchard-Jones

**Affiliations:** UCL Institute of Child Health, University College London, 30 Guilford Street, London, WC1N 1EH UK; The Institute of Cancer Research, 15 Cotswold Road, Sutton, Surrey SM2 5NG UK; Department of Pathology, Cardiff University School of Medicine, Heath Park, Cardiff, CF14 4XN UK; UCL Cancer Institute, University College London, 72 Huntley Street, London, WC1E 6BT UK

## Abstract

**Background:**

Wilms tumours (WTs) are characterised by several hallmarks that suggest epimutations such as aberrant DNA methylation are involved in tumour progression: loss of imprinting at 11p15, lack of recurrent mutations and formation of nephrogenic rests (NRs), which are lesions of retained undifferentiated embryonic tissue that can give rise to WTs.

**Methods:**

To identify such epimutations, we performed a comprehensive methylome analysis on 20 matched trios of micro-dissected WTs, NRs and surrounding normal kidneys (NKs) using Illumina Infinium HumanMethylation450 Bead Chips and functionally validated findings using RNA sequencing.

**Results:**

Comparison of NRs with NK revealed prominent tissue biomarkers: 629 differentially methylated regions, of which 55% were hypermethylated and enriched for domains that are bivalent in embryonic stem cells and for genes expressed during development (P = 2.49 × 10^-5^). Comparison of WTs with NRs revealed two WT subgroups; group-2 WTs and NRs were epigenetically indistinguishable whereas group-1 WTs showed an increase in methylation variability, hypomethylation of renal development genes, hypermethylation and relative loss of expression of cell adhesion genes and known and potential new WT tumour suppressor genes (*CASP8*, *H19*, *MIR195*, *RB1* and *TSPAN32*) and was strongly associated with bilateral disease (*P* = 0.032). Comparison of WTs and NRs to embryonic kidney highlighted the significance of polycomb target methylation in Wilms tumourigenesis.

**Conclusions:**

Methylation levels vary during cancer evolution. We have described biomarkers related to WT evolution from its precursor NRs which may be useful to differentiate between these tissues for patients with bilateral disease.

**Electronic supplementary material:**

The online version of this article (doi:10.1186/s13073-015-0136-4) contains supplementary material, which is available to authorized users.

## Background

Wilms tumour (WT) is the most common paediatric renal cancer with a prevalence of 1 in 10,000 [1]. Although a few genes that predispose to an increased risk of WT have been identified, the underlying mechanisms of Wilms tumorigenesis remain largely uncharacterised. The commonly mutated genes in sporadic WT show low mutation frequencies (*WT1* (12%) [2], *WTX* (18%) [3], *CTNNB1* (15%) [2], *DROSHA* (12%) [4], *TP53* (5%) [5]) and as most mutations often occur in the same tumour [3,6], approximately 65% of WTs are negative for all common somatic mutations. Furthermore, a recent genome-wide association study identified only two susceptibility loci of genome-wide significance and moderate effect size [7]. By contrast, up to two-thirds of WTs have abnormalities at the imprinted *IGF2*/*H19* locus on 11p15 and an epigenetic biomarker common to 118 out of 120 WTs identifiable in blood was found [8], indicating the possible involvement of epimutations such as aberrant DNA methylation [2,9]. In addition, targeted analyses identified WT-specific differentially methylated regions (DMRs) at *GLIPR1* [10], imprinted genes *NNAT* [11] and the *WT1*-antisense region [12], various satellite regions [13,14], *HACE1* [15], *RASSF1A* [16], *P16* and the protocadherin cluster at 5q31 [17].

In 40% of unilateral and almost 100% of bilateral cases, nephrogenic rests (NRs) are found juxtaposed to WTs and are considered precursor lesions [18]. NRs appear morphologically as lesions reminiscent of embryonic kidney (EK) retained from improper renal development [18]. There are two types of NR, perilobar and intralobar, that differ in terms of their location within the renal lobe and their morphological features [18]. Analysis of somatic aberrations found in WTs and their associated NRs has not clearly implicated any of the known pathways in either persistence of these presumed precursor lesions or their tumorigenic progression [19-22] and no comprehensive epigenetic analysis has yet been undertaken on NR lesions. This is largely due to the limitations of NR samples, which are microscopic lesions identified by histopathological review of formalin fixed paraffin embedded (FFPE) tissue.

Although previous studies have implicated epigenetics, embryonic or stem-like cells and disrupted renal development in WT aetiology [23,24], a comprehensive longitudinal analysis of tumour formation has not yet been undertaken. Therefore, we conducted the first longitudinal epigenetic study using NK, NR and WT trios to gain new insights into the disruption in normal renal development and the steps leading to transformation in WTs.

## Methods

### Sample collection and DNA extraction

Use of patient samples in this study was conducted with ethical approval granted by the NHS London Bridge Research Ethics Committee (reference 12/LO/0101) with experiments performed in compliance with the Helsinki Declaration. Patients included in this study were enrolled in the UK into the International Society of Paediatric Oncology (SIOP) Wilms Tumour 2001 Clinical Trial and Study (clinical trial registration number: EUDRACT 2007-004591-39) with appropriate parental consent and ethical approval. Post-nephrectomy pathology reports were studied and from those indicating the presence of NRs, FFPE blocks of the nephrectomy tissue were collected from the treatment centre. Haematoxylin and eosin-stained 3 μm sections taken from these FFPE blocks were examined independently by two paediatric pathologists who identified clearly separated regions of normal kidney (NK), NR and WT. Due to the difficulty in distinguishing between chemotherapy-treated WT and NRs in a previous study [19], sample selection was meticulous. In total 36 NKs, 24 NRs (5 intralobar NRs and 19 perilobar NRs) and 37 WTs were identified including a total of 23 matched trios. Microdissection was carried out by either a 2 μm core sample (for blocks composed entirely of NK or WT) or by cutting 20 to 30 5-μm sections (dependent on region area) and removing the desired tissue with a scalpel. DNA extraction from FFPE tissue was carried out using the DNeasy Blood & Tissue Kit (QIAGEN, Hilden, Germany). Manufacturer’s instructions were modified with an additional 90°C heating step for 1 hour after overnight incubation at 56°C and a 10 minute incubation at 70°C after adding AL buffer.

### Genome-wide methylation analysis using Illumina 450 k BeadChips

An optimised FFPE protocol was followed [25] whereby 0.5 to 2 μg DNA (depending on available yield) was treated using the REPLIg FFPE kit (QIAGEN) and the EZ DNA Methylation kit (Zymo Research Corp, CA, USA). Methylation-specific primers were used to confirm bisulfite-conversion success of at least 98%. A total of 97 samples were profiled using the Illumina Infinium 450 k platform [26]; these were processed by UCL Genomics according to manufacturer’s instructions. The scanned BeadChip microarray data were interpreted by GenomeStudio software (v1.9.0, Illumina) and then analysed using R statistical software v3.02 [27]. Prior to statistical analyses, data were filtered to remove samples with low coverage and poor density profiles, resulting in the exclusion of one NK, two NR and one WT sample, leaving a total of 20 matched trios. Further quality control and data normalisation using the Bioconductor package ChAMP [28] implemented the removal of all probes where at least one sample showed poor detection (detection *P* > 0.01), leaving 435,385 normalised β-values. The 450 k methylation data described in this study are available from the Gene Expression Omnibus with accession ID GSE59157.

### Statistical analysis of methylation β-values

All statistical analyses were performed using R. To make comparisons between tissues, the Bioconductor package Limma [29] was used to generate a Bayesian framework linear model that performed three-way contrasts between the tissue types for the ANOVA analysis. For comparison of two tissue types, both histology type and patient were considered in a Bayesian model which made intra-patient comparisons at each CpG and then compared these across all patients to generate average Δβ values with corresponding *P*-values which were corrected for multiple testing using the Benjamini-Hochberg model [30]. DMRs were identified using the Probed Lasso algorithm implemented through the Bioconductor package ChAMP [28]. This algorithm uses the Illumina annotation to identify the nearest neighbour CpG for every probe and generates a category-specific average probe density based on the CpG location. It therefore considers the non-uniform distribution of probes across the genome with large between-probe distances seen at intergenic regions and small distances seen at TSS200 regions. From setting the minimum lasso size to 10 bp, the algorithm calculates the respective probe-lasso size within each category and centres this lasso at each probe. Next, using the output topTable from Limma, only those probes with a false discovery rate (FDR) <0.01 were called significant. DMRs were then defined if the lasso connected three or more significant probes. Any non-significant probes within the lasso region were also included in the DMR to better gauge DMR significance and those DMRs within 1 kb of each other were encompassed into one region.

To compare tissues avoiding cell type composition effects, the RefFreeEWAS algorithm was applied [31]; this uses single value decomposition to estimate the number of cell types contributing to overall histology. In this study, the number of cell types contributing to methylation signal was estimated as d = 3. Using this parameter, the algorithm deconvoluted the β-values using a design matrix specifying patient pairs and sample histology, and generated bootstrap-derived CpG-specific *P*-values (not corrected for multiple testing) and covariates that correspond only to a ‘phenotype-specific’ methylation signal with no cell mixture effects as previously described [32].

To compare variance among groups, a Bartlett’s test was run using R. Probe-wise comparisons were made to assess the difference in variance between groups. Embryonic stem cell (ESC) chromatin data were extracted from Gene Expression Omnibus/NCBI (accession ID GSE8463). Enrichment of epigenetic or genetic features was determined by comparison of significant CpGs against an equal sized cohort generated by multisampling all 450 k array probes present after normalisation. To identify the frequency of tumours with hypermethylated tumour suppressor genes, tumours were classed as hypermethylated if the average β-value for all CpGs in a DMR was greater than the average for the NR cohort plus 1 standard deviation. Pathway and gene ontology process analysis was conducted using GREAT [33] with all CpGs present after normalisation used as a reference file. Processes with a significant fold enrichment (>2) were selected with the Bonferroni corrected *P*-value <0.01 and with at least four significant genes per pathway.

### Comparison with embryonic kidney

Human EKs were provided by the Joint MRC/Wellcome Trust Human Developmental Biology Resource at the UCL Institute of Child Health. Ethical approval was covered by the HDBR HTA tissue bank license and project approval. Details of approval terms can be found at [34]. DNA was extracted from four human EKs (from gestational age 8 weeks and 12 weeks) using the AllPrep DNA/RNA Micro Kit (QIAGEN) according to the manufacturer’s instructions. Methylation levels for EK were derived using 450 k BeadChips as described above. The 20 trios plus 4 EKs were re-normalised together including a between-array normalisation using wateRmelon package Dasen to correct for between-array effects [35]. The final dataset included 330,731 CpGs with probes that map to sex chromosomes, with known SNPs at the target site or that bind multiple genomic loci (defined from *in silico* analyses [36]) excluded. Comparisons between tissues were performed using Limma and RefFreeEWAS with an unpaired design.

### Validation by bisulfite sequencing

In total, 5 regions were selected for validation, which covered 18 CpGs interrogated by the 450 k array. Primers were designed for bisulfite-converted DNA using MethPrimer [37]. In total, 10 ng of bisulfite-converted FFPE extracted DNA from four trios was used to amplify the specific genomic regions. The hot-start enzyme KAPA HiFi Uracil + (KAPA Biosystems Inc, Wilmington, MA, USA) was used for PCR and products were cleaned using magnetic beads (Beckman Coulter Inc, Brea, CA, USA) and quantified using Picogreen reagents. Samples were tagged and pooled prior to sequencing on the Illumina MiSeq according to the manufacturer’s instructions.

Raw MiSeq paired-end reads were mapped to human genome build hg19 with Bismark v0.9.0 [38] using Bowtie 2 [39] as the aligner. Methylated and unmethylated base counts were generated with the bismark_methylation_extractor utility and exported as BedGraph files for further analysis and display in Integrative Genomics Viewer [40]. Aligned BAM files were sorted and indexed with SAMtools [41] for assessment of the regions of interest in Integrative Genomics Viewer. The number of C reads (methylated prior to conversion) was divided by the total number of reads per bisulfite-sequenced CpG site to discern the percentage methylation. These were then compared with the respective 450 k β-values to compare platforms.

### RNA sequencing

RNA was extracted from 12 samples (4 trios) by cutting multiple 5 μm sections of FFPE tissue and scraping the target region using a new sterile scalpel blade each time. Tissue was put into an Eppendorf then RNA was extracted using the RNeasy FFPE kit (QIAGEN) according to the manufacturer’s instructions. Library preparation for the 12 samples was performed using the TruSeq RNA access kit (Illumina) and run on the Illumina NextSeq 500. Reads were aligned using TopHat2 [42] and counted using HTseq [43] in Python. Two samples were excluded from the analysis due to poor read coverage and aberrant clustering in unsupervised analysis. The Bioconductor package DESeq [44] was used in R to make group-wise comparisons between NK and NR, then NR and WT, run with default parameters.

## Results

### Methylation profiles distinguish tissue types and show increased variability in both NR and WT samples compared to NK

**Figure 1:**
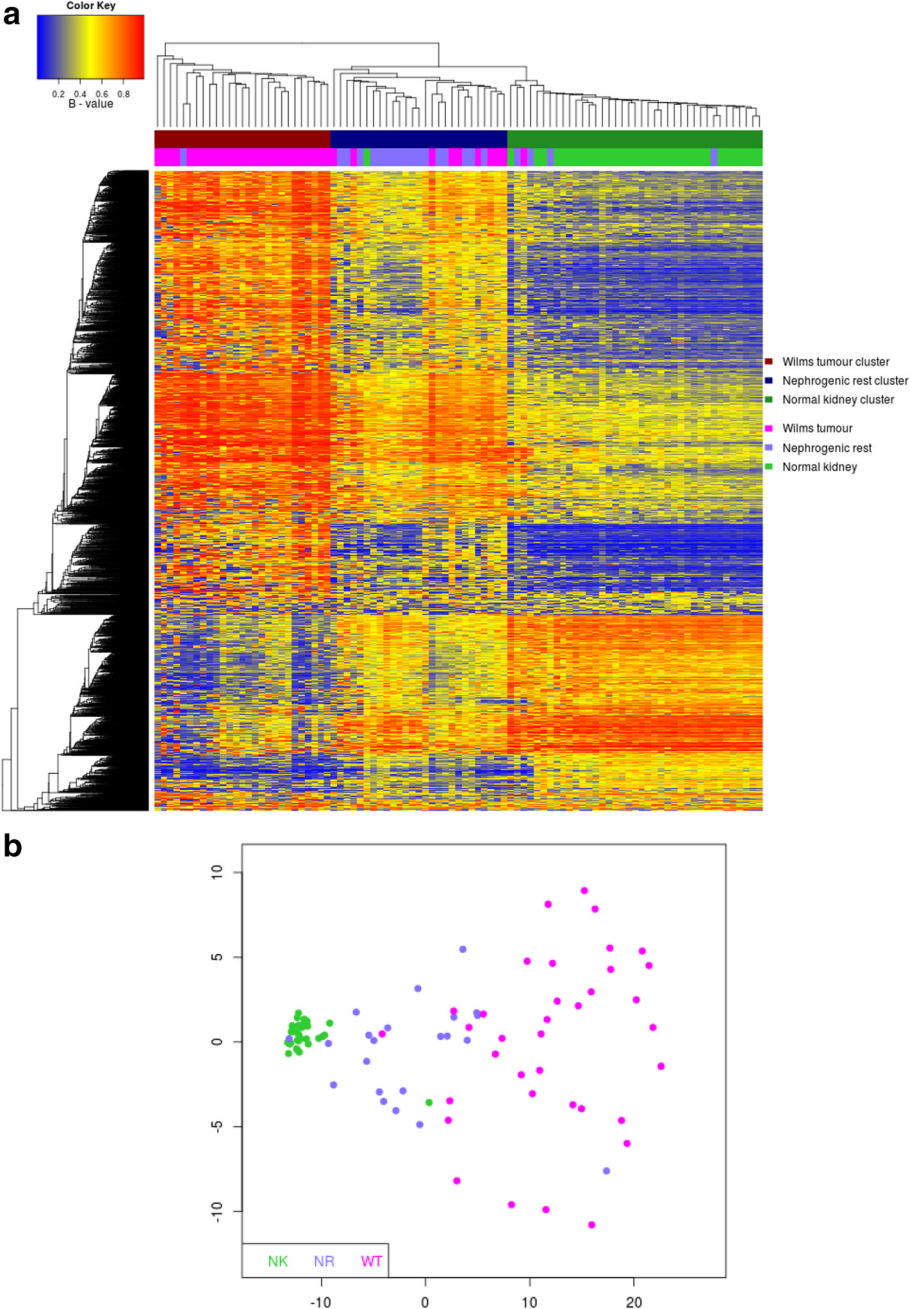
**Unsupervised analysis of methylation values in normal kidney (NK), nephrogenic rests (NR) and Wilms tumour (WT). (a)** Unsupervised consensus clustering of the top 1% most variable positions across the full dataset as determined based on interquartile range. Three clusters were formed which predominantly separated tissue types. The 'Wilms tumour cluster' (dark red) is WT-predominant with 26 WT (pink) and 1 NR (blue) sample, which is separated from the 'nephrogenic rest cluster' (navy) with 17 NR, 9 WT and 1 NK (green) sample and the 'normal kidney cluster' (dark green) with 34 NK, 4 NR and 1 WT sample. As the nephrogenic rest cluster contains several WT samples, some tumours may not be as epigenetically distinct from their precursor lesions as suggested by their morphology. **(b)** Multidimensional scaling of the top 1% most variable positions showed greater variability across the NR and WT datasets compared with NK.

To characterise tissue-specific methylation changes for NK (n = 35), NRs (n = 22) and WTs (n = 36; including 20 matched trios) we derived methylation levels (β; 0 = unmethylated, 1 = methylated) for 435,385 CpGs using Illumina Infinium HumanMethylation450 BeadChips and validated β-values using bisulfite-sequencing, which showed good concordance (R = 0.8365, with a median difference in β-value of 0.09; Figure S1 in Additional file 1; Additional file 2). Unsupervised clustering of the 1% most variable CpGs (excluding probes that map to sex chromosomes or with known SNPs at the target site; termed XYS probes) revealed clear separation of samples into tissue-related groups (Figure 1a), confirming the significant association between tissue type and methylation. Although both intralobar NRs (n = 5) and perilobar NRs (n = 17) were present, unsupervised analysis did not distinguish between them and as sample groups were small, we did not interrogate for further differences. Next, we performed multidimensional scaling of the top 1% most variable CpGs to assess the inter-sample variability within each dataset. The NK samples grouped tightly together; however, the NR and WT datasets both showed high variability as illustrated by the wide dispersion of data points (Figure 1b). Furthermore, we found that for probes showing significant non-homogeneity of variances (Bartlett test), the vast majority exhibited increased variance in NR and WT groups compared with the NK group (N_NK>NR_ = 9,334; N_NR>NK_ = 94,546 and N_NK>WT_ = 14,933; N_WT>NK_ = 158,189; Figure S2 in Additional file 1).

### Supervised analysis reveals two Wilms tumour groups

**Figure 2:**
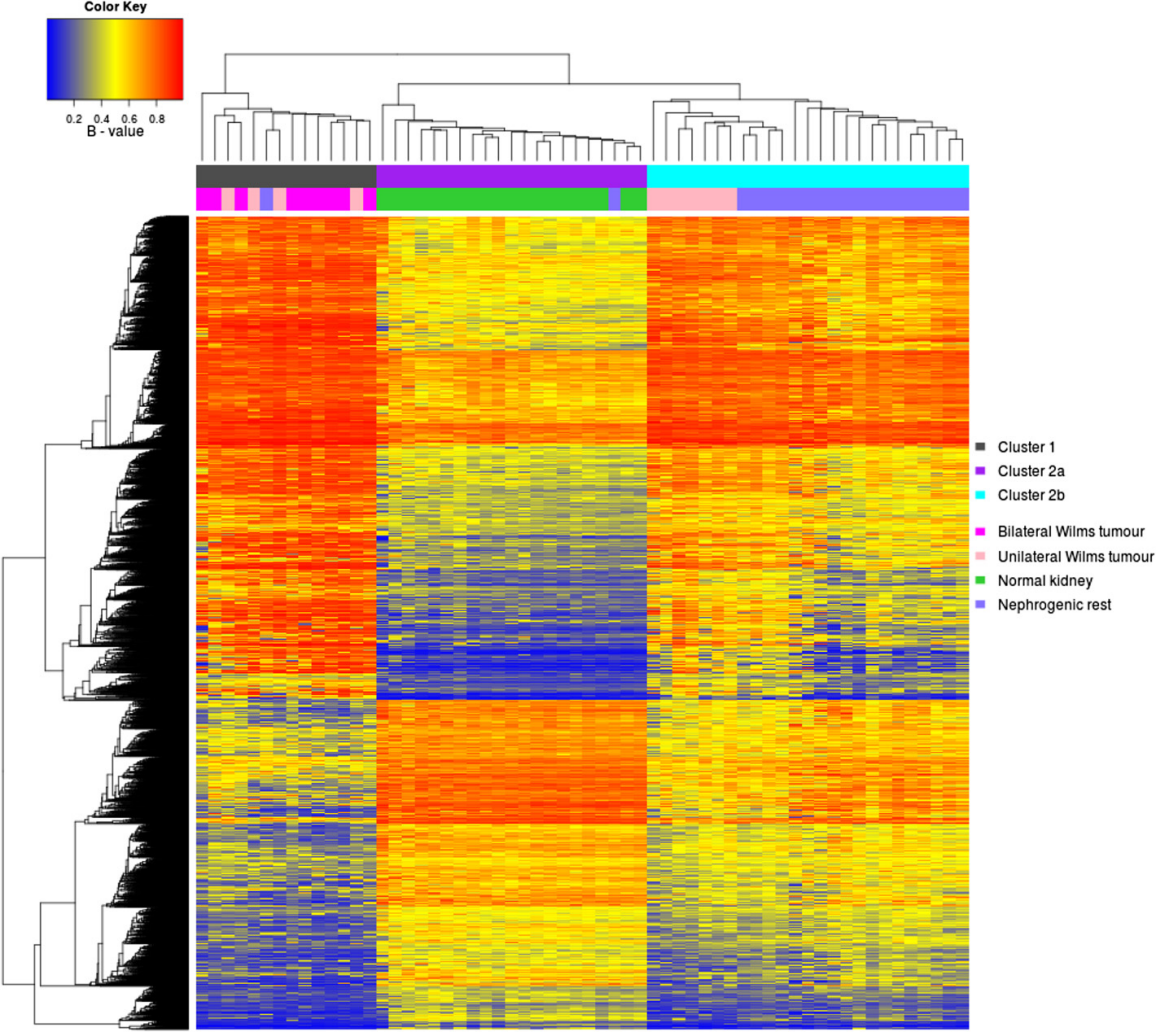
**ANOVA analysis identifies two Wilms tumour groups.** Consensus clustering of the significant CpGs (n = 7,921; *P* < 5 × 10^-8^) from ANOVA analysis of 20 trios of normal kidney (green), nephrogenic rest (blue) and Wilms tumour (pink). Here, three clusters can be seen which show the presence of two distinct WT groups. Cluster 1 (grey) comprises group-1 WT (n = 13), which includes all bilateral WT (dark pink) and 4 unilateral cases (light pink). Group-1 WT clusters separately from their associated NRs. The second cluster further separates into two, with cluster 2a (purple) containing all NK samples and cluster 2b containing group-2 WTs (n = 7), which are all unilateral and cluster together with their associated NRs.

As methylation status clearly distinguished between tissue types (NK, NR and WT), we focussed on the set of 20 matched trios (clinical information in Additional file 3) and performed ANOVA on the full dataset with XYS probes excluded to identify CpGs that were differentially methylated between all three tissue types. This analysis identified 7,921 CpGs reaching genome-wide significance (*P* < 5 × 10^-8^). Upon clustering of these CpGs, two clusters formed: cluster 1 (13 WT, 1 NR) and cluster 2, which further separated into cluster 2a (20 NK, 1 NR) and cluster 2b (7 WT, 18 NR; Figure 2). All NK samples fell into cluster 2a but the WT samples fell into two distinct groups. Cluster 1 WT (termed group-1 WT) appeared distinct from their NR whereas cluster 2b WT (termed group-2 WT) clustered with their respective precursor lesion. Upon further investigation, we observed that all WTs from patients with bilateral disease fell into group-1, giving a significant association between distinction from NRs and bilateral disease (*P* = 0.032, chi-square test). This was further supported by re-evaluation of the unsupervised multidimensional scaling analysis where group-2 WT appeared closer to NR samples (Figure S3 in Additional file 1). As this multidimensional scaling plot showed a wider dispersion of group-1 WTs, a Bartlett test was performed to compare levels of probe-specific variance between group-1 and group-2 WTs. This test showed that group-1 WTs had 2.4 times as many probes with a significant increase in variance compared with group-2 WTs (31,638 compared with 13,124; *P* < 0.01), suggesting that group-1 WTs have a more hypervariable epigenome.

To further investigate whether two WT groups exist that differ in terms of relationship with their respective NRs, we separated group-1 (n = 13) and group-2 (n = 7) WT-NR matched pairs and used a paired linear model to identify intra-patient sites of differential methylation that were common across samples. The matched study design avoids patient-specific SNPs from giving false positives and XYS probes were thus included. For group-1 WT we identified 22,344 methylation variable positions (MVPs; FDR <0.01). Conversely, group-2 WTs showed no significant sites of differential methylation compared with their associated NRs.

### Wilms tumour cells show hypomethylation of key renal development genes and silence tumour suppressor genes by hypermethylation

Next, group-1 MVPs were grouped into discreet clusters to further investigate their biological relevance [28]. In total, 625 DMRs were identified, of which 460 (73.6%) were hypomethylated and 165 (26.4%) were hypermethylated in WTs with respect to NRs; termed hypo-WT-DMRs and hyper-WT-DMRs respectively. Hyper-WT-DMRs were smaller and were more often located at transcription start sites and within CpG shores, indicating a relationship with tissue identity as well as gene expression [45,46] (Table S3 in Additional file 1). Conversely, hypo-WT-DMRs were enriched within gene bodies and were not associated with CpG islands, shores or shelves. By interrogation with GREAT [33], which associates genomic positions with gene regulatory domains to infer biological significance, we found that hypo-WT-DMRs were enriched within developmental processes, including metanephric nephron development and nephron development involving genes such as *GDNF*, *IRX2*, *PDGFB*, *POU3F3* and *SOX8* and in processes involved in stem cell maintenance, development and differentiation (Table S4 in Additional file 1). Conversely, hyper-WT-DMRs were enriched for genes involved in cell adhesion processes and processes associated with regulation of transcription (Table S5 in Additional file 1).

To demonstrate the effect on gene expression, RNA sequencing was performed on four trios. Comparison between NR and WT identified 75 genes with significant differential expression (FDR <0.05) including genes involved in cell adhesion (*CD200*, *GPR108*, *TSPAN2*, *ADAMTS8*, *MDK* and *NCAM1*) and in regulation of transcription (*NFKB1*, *MYSM1*, *PREPL*; Table S6 in Additional file 1). These data support the dysregulation of these processes during progression from the precursor lesion, as identified by interrogation of hyper-WT-DMRs. *NCAM1* has previously been identified as being a marker for cancer-propagating WT cells [47], suggesting its potential as a marker of transformation from NRs.

To identify methylation changes associated with transformation, we studied the hyper-WT-DMRs further to see whether we could link hypermethylation with tumour suppressor gene silencing. Of the 123 genes associated with the 165 hyper-WT-DMRs, 5 were found within TSgene, the Tumour Suppressor gene database [48] and we predicted they would be inactivated in group-1 WT (Table 1). Indeed, RNA sequencing showed downregulation of *CASP8*, *RB1* and *TSPAN32* in WTs compared with NRs (Table 1); however, due to small sample numbers, these differences did not reach statistical significance. As *MIR-195* and *H19* are a miRNA and a non-coding RNA, respectively, these were not detected by this assay. Of these, *H19* DMR methylation (and hence presumed loss of imprinting) has been previously reported in approximately 70% all WTs [2]. Here we see hypermethylation in 85% NR-associated group-1 WT as an event associated with transformation. For the 11 of 13 WT samples with gain of methylation at *H19*, NK showed average methylation levels lower than NRs (0.70 versus 0.78), both of which were significantly lower than WTs (0.88, *P* = 5.6 × 10^-7^ and *P* = 3.1 × 10^-6^, respectively), suggesting that, although a major increase in methylation occurred upon transformation, NK may contain a proportion of cells with methylated *H19* DMR as this imprinted region showed higher than expected methylation levels.

**Table 1:** **Tumour suppressor genes hypermethylated in group-1 Wilms tumours**

**Gene**	**DMR** ***P*** **-value**	**Hypermethylated WT**	**Average reads in NR**	**Average reads in WT**
*CASP8*	0.0037	10 of 13	104	20
*H19*	0.0045	11 of 13	NA	NA
*MIR195*	0.0049	13 of 13	NA	NA
*RB1*	0.0020	13 of 13	55	11
*TSPAN32*	0.0091	10 of 13	18	8

### Cell composition correction identifies 'pheno-MVPs'

Although the MVPs identified and described here are valid tissue biomarkers that distinguish NRs from WTs encompassing the heterogenous nature of each tissue, these findings may also be due to the known variable cell type composition (as shown in Figure S4 in Additional file 1). To take this into consideration, the RefFreeEWAS algorithm was applied to the 20 NR-WT pairs. This algorithm uses single value decomposition to identify changes in methylation associated with a cell mixture, providing adjusted covariates and *P*-values that represent direct epigenetic effects [31]. Such pheno-MVPs, as previously described [32], most accurately reflect phenotypic methylation changes. In total, 37,118 pheno-MVPs were identified (*P* < 0.01). Of these, 12,929 (35%) were hypermethylated and 24,189 (65%) were hypomethylated in WTs with respect to NRs. As a cell-type composition corrected β-value matrix cannot be generated by this package, and no algorithm for DMR detection is included, we cannot comment on whether the two groups or the biomarker DMRs were detected as a result of cell composition effects. Instead, we compared the MVPs identified by each respective method and found that 9,651 (36%) of MVPs identified by the non-corrected Limma algorithm were also detected by RefFreeEWAS. Genes with the largest number of pheno-MVPs included *ARHGEF16*, *SIM2*, *H19*, *GALNT5*, *U6*, *ALG10*, *IRX4*, *TBX15*, *VAX2*, and *PRRT1* and significantly overlapped with genes showing polycomb-associated H3K27me3 in normal tissue that gained methylation in cancer tissue [49] (*P* = 9.11 × 10^-126^; 246 CpGs, identified using GREAT).

### Aberrant hypermethylated DMRs in NR tissue suggest developmental arrest

After demonstrating the presence of two WT groups according to the epigenetic relationship to their associated NRs, we next focused on characterising the NR methylome. There was no evidence of differences between NRs as 18 out of 20 fell into ANOVA cluster 2b (Figure 2). Therefore, we performed linear modelling on the 20 NK-NR pairs to identify methylation changes associated with incomplete renal development. The comparison between NK and NR identified 23,667 differentially methylated MVPs (FDR <0.01), which were grouped into 629 DMRs with relatively equal proportions of hyper- and hypomethylation (55% and 45%, respectively). We termed these kidney-rest DMRs (KR-DMRs; Table S7 in Additional file 1) with hypo-KR-DMR and hyper-KR-DMR referring to hypomethylation and hypermethylation in the NRs with respect to NK. Analysis of hypo-KR-DMRs did not result in overrepresentation of any processes that could be readily associated with developmental arrest (Table S8 in Additional file 1); however, analysis of hyper-KR-DMRs, which were significantly enriched within CpG shores (9.9%, empirical *P*-value = 0.01), showed overrepresentation of developmental or multicellular organismal processes (Table S9 in Additional file 1). The overrepresented processes included early embryonic patterning, and we hypothesised that gain of methylation may be occurring at developmental loci required to complete nephrogenesis.

**Figure 3:**
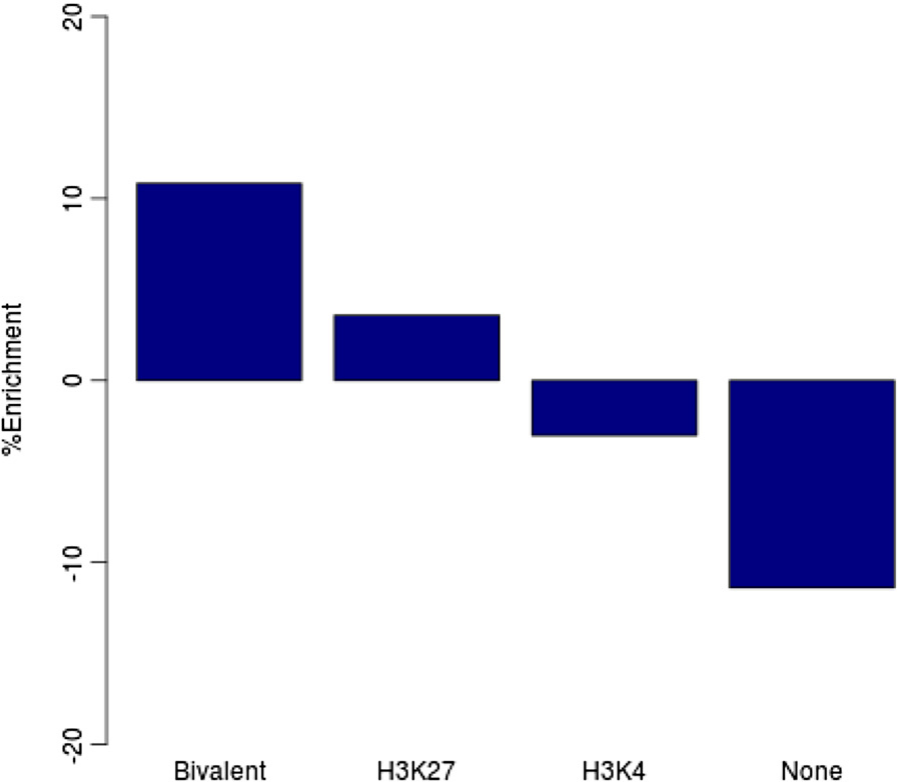
**Hypermethylated KR-DMRs are enriched in developmental loci and genes involved in β-catenin localisation.** Hypermethylated KR-DMRs showed 10.8% enrichment (empirical *P* ≤ 0.01) for location within domains that are bivalent in embryonic stem cells (considered as developmental loci) compared with levels ascertained by repeated multiple sampling of all array CpGs.

To test this hypothesis, we explored the overlap between the hyper-KR-DMRs and regions of active chromatin (with chromatin mark H3K4me3) and regions of repressed chromatin (with H3K27me3) in ESCs [50,51]. In ESCs, the combination of both marks (bivalent domains) allows for loci to be poised in a state awaiting differentiation signals that either rapidly repress or express the underlying gene. Multisampling analysis revealed a strong, significant enrichment of hyper-KR-DMRs within bivalent domains (10.8%, empirical *P* = 0.01; Figure 3; by comparison, hypomethylated KR-DMRs were negatively enriched by -1.9%). As bivalent domains mark key developmental genes poised for differentiation, this positive enrichment suggests that DNA hypermethylation may contribute to the developmental arrest seen in NRs.

To take into consideration cell composition effects, the RefFreeEWAS algorithm was also applied to the comparison between 20 NR-NK pairs. This analysis identified a total of 61,497 pheno-MVPs with 28,495 (46%) hypo- and 33,002 (54%) hypermethylated in NR with respect to NK. In total, 69% non-corrected MVPs overlapped with the pheno-MVPs. These pheno-MVPs also showed a significant association with genes that are H3K27me3 marked by polycomb proteins in normal tissue that acquire cancer-specific methylation [49] (*P* = 1.76 × 10^-20^; 141 CpGs).

### Comparison with embryonic kidney shows aberrant gain of methylation at Polycomb sites is not associated with developmental stage

As the WT cell-of-origin is embryonic, methylation levels were compared between EK (n = 4), NRs and WTs. For these analyses, as we do not need to identify tissue-specific biomarkers and the EK was not matched, each comparison was performed using the RefFreeEWAS algorithm [31]. To begin with, we focussed on the pheno-MVPs that differentiate between NRs and EK. As previously mentioned, it was not possible to generate DMRs using the RefFreeEWAS package and we therefore focussed on pheno-MVPs with *P* < 0.01 and Δβ > |0.2|. Of the 4,457 MVPs identified in this comparison, 2,108 were hypo-MVPs and 2,349 were hyper-MVPs in NRs with respect to EK. Although MVP selection here was different from the previous DMR selection, similarly to the NR-NK comparison, many of the hyper-MVPs fell within key genes involved in renal development and were therefore enriched in renal development processes. Hyper-MVPs also showed a significant association with regions identified as Polycomb repressive complex 2 (PRC2) targets in ESCs (*P* = 2.79 × 10^-66^) [52], including a set of 189 genes and 480 CpGs (20% of hyper-MVPs). This concordance of results between comparisons of NRs with each of NK and EK suggests that the gain of methylation observed here is a true aberrant event associated with NR formation rather than an epigenetic feature reminiscent of an early developmental stage.

Next, we focussed on pheno-MVPs identified from comparison of WTs with EK, of which 5,814 (44%) were hypomethylated and 7,538 (56%) were hypermethylated in WTs with respect to EK. We first focussed on WT hyper-MVPs and, by interrogation with GREAT, identified similar developmental processes as identified in the NR-EK comparison, suggesting maintenance of the epigenetic landscape from the NR. The surprising difference was that the WT-EK comparison highlighted many more processes involved in general embryonic development instead of specifically renal development, including, for example, 228 genes involved in embryonic pattern specification and 251 genes involved in embryonic morphogenesis (the most differentially methylated included *FOXD1*, *GLI2*, *HOXA5*, *HOXD10*, *LBX1*, *PAX2*, *SIM2*, *SIX3*, *TBX3*, *UNCX*, *VAX2* and *WNT10A*). Furthermore, a significant enrichment was again seen for hyper-MVPs within regions of PRC2 binding (*P* = 3.92 × 10^-217^), but there was also a very significant enrichment for regions of H3K27me3 (*P* = 2.91 × 10^-247^), Polycomb EED targets (*P* = 1.08 × 10^-241^) and Suz12 targets (*P* = 8.65 × 10^-207^), all identified by ChIP on chip in human ESCs [52]. This evidence suggests a further dysregulation of methylation at Polycomb target sites and developmental loci as cells progress towards malignancy.

## Discussion

In this study, we show that regional differences in DNA methylation can discriminate between NK, NRs and WTs. We highlight that both NRs and WTs have more between sample variability than NK with increased variability associated with tumourigenesis, a finding consistent with adult adenocarcinoma of the colon [9]. In this study, NR formation, by comparison with NK and EK, was associated with hypermethylation of genes involved in renal development and loci that show bivalent chromatin marks in ESCs. Although this enrichment at bivalent domains suggests that DNA hypermethylation may contribute to the developmental arrest seen in NRs, recent evidence [53] suggests that bivalent marking is more ubiquitous than previously thought, thus potentially reducing its specificity as a marker for the poised state, if confirmed. These same loci were PRC2 target sites that show H3K27me3 in normal tissue and are commonly methylated in other cancers. These similar findings, in both non-corrected and corrected analyses for cell type composition and in comparison with both NK and EK tissues, suggests that the initiating step in Wilms tumourigenesis - that is, NR retention in post-natal kidney - involves PRC2-associated gain of methylation (either by an active or passive mechanism) at renal development loci required for normal nephrogenic differentiation, which is not cell composition-mediated. NRs cannot, therefore, differentiate normally and remain as aberrant embryonic-like tissue in the post-natal kidney. Polycomb target hypermethylation has previously been associated with the cancer phenotype and less well-differentiated tumours [52]. It has been proposed that the disruption of normal Polycomb mechanisms is central to tumour initiation [54], and gain of methylation has been detected in pre-malignant lesions for other adult cancers [55].

Supporting the role of Polycomb protein dysregulation in WTs, evidence from a mouse model of *in vivo* reprogramming associated formation of WT-like lesions with failure of Polycomb gene targets to be repressed [56]. Furthermore, upregulation of Polycomb genes *BMI-1*, *EZH2*, *SUZ12* and *EED* was seen in progressive blastemal-enriched WT xenografts in mice, suggesting their expression correlated with tumourigenesis [57]. The question that remains is what causes PRC overexpression in the first place? Genetic mutation could be involved and DNA sequencing projects are currently underway that may highlight novel mutations in WTs associated with Polycomb gene regulation.

This study presents novel evidence that WTs with associated NRs fall into two distinct subsets according to whether they have a similar (group-2) or distinct (group-1) methylome. We hypothesise that group-2 WTs may be driven by somatic mutation and have a more stable epigenome that remains close to that of their precursor NR as no significant common changes in methylation occur between WTs and NRs. Furthermore, as group-1 WTs significantly associate with bilateral disease, we predict that the event leading to NR formation occurs at an earlier time point in embryogenesis as both kidneys are affected. We therefore hypothesise that the progenitor cells within this population are more epigenetically unstable, regardless of their association with potentially epigenome-modifying genetic mutations, which results in hypermethylation of tumour suppressor genes, giving selective advantage and causing transformation. *CASP8* and *H19* have been previously associated with WTs [58,59], and H19 in particular has been associated with sporadic bilateral disease [2], whereas *RB1*, *Mir-195* and *TSPAN32* aberrations have not previously been identified in WTs, although detected in other cancers [60-68]. This epigenetic plasticity will be replicated in the tumour-initiating cell, which would allow the resultant proliferating tumour to evolve into an entity with a distinct epigenetic profile from the NR. This is supported by evidence showing that group-1 tumours have a greater number of significantly more variable probes than group-2 tumours. In group-1 WT we saw hypomethylation of genes that, if expressed as predicted, give WTs an EK-like profile similar to that observed in previous WT chromatin and expression profiling studies [23,24]. This study shows that obtaining this phenotype is associated with the stage of transformation and not with the precursor lesion. Also associated with transformation was gain of methylation at *H19*. The *H19* DMR showed high levels of methylation in both NK and NRs, but levels significantly increased upon transformation to WTs, which was not confounded by cell type composition.

## Conclusions

Methylation profiles vary significantly between NK, NRs and WTs and changes in the methylome underlie both NR formation and transformation to WTs in a subset of cases. We have presented the first molecular association between developmental arrest and NR formation and showed the presence of two distinct WT groups by methylome comparison with their associated NRs. These genome-wide and gene-specific assays, which work well on formalin-fixed tissue, have potential clinical utility to distinguish more accurately between NRs and treated WTs in patients with bilateral disease. This distinction, which is often difficult to make unambiguously by histological examination, would be useful for post-operative treatment planning (determining whether the resection margin is clear of tumour, which dictates the need for radiotherapy) and would aid in the evaluation of the efficacy of nephron-sparing surgery in achieving complete tumour excision. However, the potential use of a molecular marker for this purpose requires validation in an independent set of cases. Finally, as group-1 tumours appear more epigenetically unstable, we propose that epigenetic modifiers be considered as candidate therapeutic targets for WT and prevention of NR transformation in pre-disposed individuals, particularly as few targeted therapies have emerged to date based on somatic mutational analysis.

## Additional files

Additional file 1: Figure S1. Validation of the 450 k array using bisulfite-sequencing. **Figure S2.** Probe-wise variance between groups shows NRs and WTs are much more variable than NK. **Figure S3.** Unsupervised analysis shows two WT groups. **Figure S4.** Histological composition of microdissected tissue. **Table S3.** Mutation analysis of *WT1*, *WTX* and *CTNNB1* in WT. **Table S4.** Description of hypermethylated and hypomethylated differentially methylated regions (DMRs) in group-1 WT compared with matched nephrogenic rests. **Table S5.** Significantly overrepresented biological processes identified by hypomethylated WT-DMRs. **Table S6.** Significantly overrepresented biological processes identified by hypermethylated group-2 WT-DMRs. **Table S7.** Description of hypermethylated and hypomethylated differentially methylated regions (DMRs) in nephrogenic rests compared with matched normal kidney. **Table S8.** Significantly overrepresented biological processes identified by hypomethylated KR-DMRs. **Table S9.** Significantly overrepresented biological processes identified by hypermethylated KR-DMRs. Additional file 2: Table S1. Comparison of 450 k β-values and levels of methylation determined by bisulfite sequencing. Additional file 3: Table S2. Clinical information for the patients from which the 20 matched trios originated.

## Abbreviations

DMR: differentially methylated region
EK: embryonic kidney
ESC: embryonic stem cell
FDR: false discovery rate
FFPE: formalin fixed paraffin embedded
H3K4me3: histone 3 lysine 4 trimethylation
H3K27me3: histone 3 lysine 27 trimethylation
MVP: methylation variable position
NK: normal kidney
NR: nephrogenic rest
PCR: polymerase chain reaction
WT: Wilms tumour
