# Supplementary material for: Comparative methylome analysis identifies new tumour subtypes and biomarkers for transformation of nephrogenic rests into Wilms tumour
Source: Genome Med. 2015 Feb 2;7(1):11. doi: 10.1186/s13073-015-0136-4 (PMC4354990; doi:10.1186/s13073-015-0136-4)
Supplement: Additional file 1: Figure S1. — Validation of the 450 k array using bisulfite-sequencing. Figure S2. Probe-wise variance between groups shows NRs and WTs are much more variable than NK. Figure S3. Unsupervised analysis shows two WT groups. Figure S4. Histological composition of microdissected tissue. Table S3. Mutation analysis of WT1, WTX and CTNNB1 in WT. Table S4. Description of hypermethylated and hypomethylated differentially methylated regions (DMRs) in group-1 WT compared with matched nephrogenic rests. Table S5. Significantly overrepresented biological processes identified by hypomethylated WT-DMRs. Table S6. Significantly overrepresented biological processes identified by hypermethylated group-2 WT-DMRs. Table S7. Description of hypermethylated and hypomethylated differentially methylated regions (DMRs) in nephrogenic rests compared with matched normal kidney. Table S8. Significantly overrepresented biological processes identified by hypomethylated KR-DMRs. Table S9. Significantly overrepresented biological processes identified by hypermethylated KR-DMRs. [file 13073_2015_136_MOESM1_ESM.docx]

**Supplementary Information for:**

**Comparative methylome analysis identifies new tumour subtypes and biomarkers for transformation of nephrogenic rests into Wilms tumour**

Jocelyn Charlton^1^, Richard D. Williams^1^, Neil J. Sebire^1^, Sergey Popov^2^, Gordan Vujanic^4^, Tasnim Chagtai^1^, Mariana Maschietto^1^, Marisa Alcaide-German^1^, Tiffany Morris^3^, Lee M. Butcher^3^, Paul Guilhamon^3^, Stephan Beck^3,^*, Kathy Pritchard-Jones^1,^*

**Figure S1: Validation of the 450k array using bisulfite-sequencing**

The β-values discerned by the 450k array (x-axis) compared to level of methylation detected using bisulfite-sequencing (y-axis) show good correlation (Pearson correlation coefficient = 0.8365).


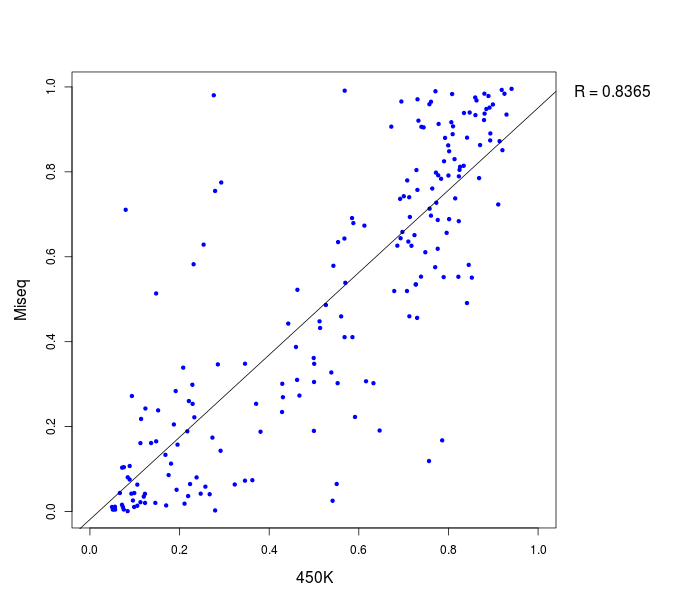


**Figure S2: Probe-wise variance between groups shows NR and WT are much more variable than NK**

Variance within the NK group was consistently lower compared to NR and WT groups. Blue bars show observed data, while the red line indicates the null distribution.  We performed Bartlett tests to identify probes showing significant (p < 0.01) departures from homogeneity of variance between groups. Each time we found the NK group was substantially skewed toward decreased variability compared with NR groups (skew = -87.89, p < 2.2x10^-16^, D'Agostino skewness test; Fig. S3a) and WT groups (skew = -160.10, p < 2.2x10^-16^, D'Agostino skewness test; Fig. S3b).

**a**

**b**

**
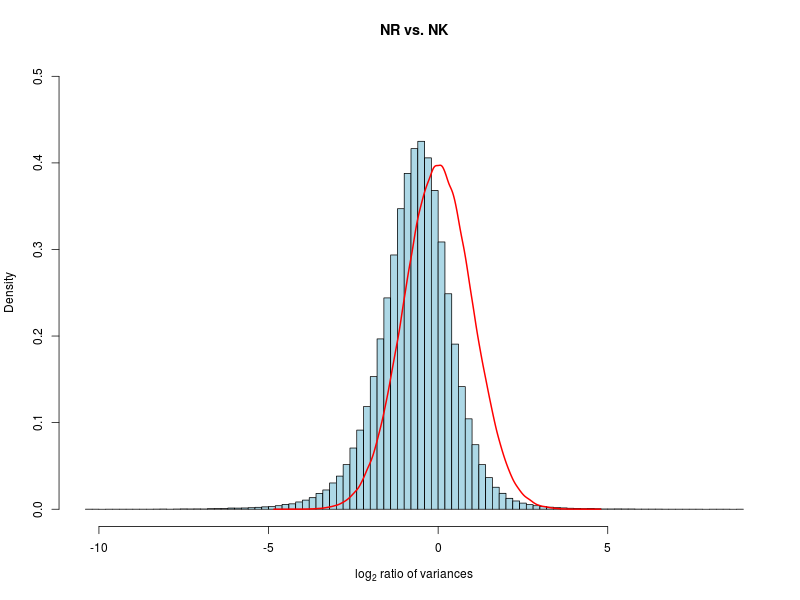
**

**
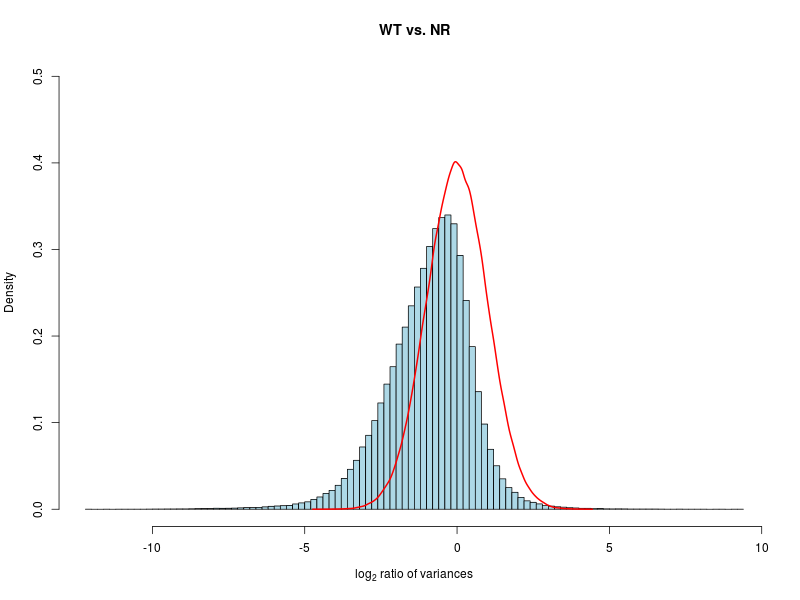
**

**Figure S3: Unsupervised analysis shows two Wilms tumour groups**

Re-evaluation of tumour separation by multidimensional scaling of the top 1% most variable positions showed separation of the two Wilms tumour groups 1 (orange) and 2 (red). Group 2 are more intermixed with the NR samples whereas group 1 are located further away although wide variance is seen.


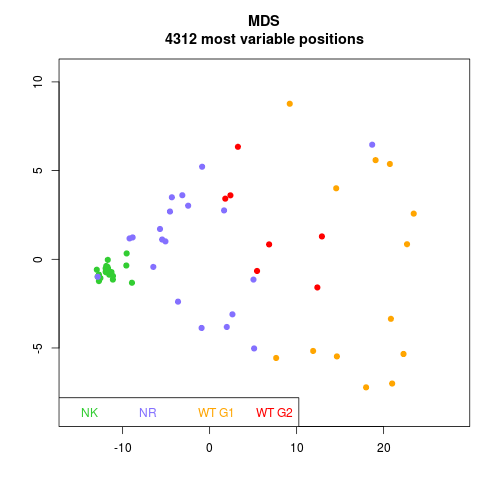


**Figure S4: Histological composition of microdissected tissue**

Proportions of each cell type (blastema (navy), stroma (green), epithelia (pink) or necrosis/chemotherapy induced changes (CIC; yellow)) within each microdissected nephrogenic rest (NR) or Wilms tumour (WT) section. Samples are ordered by patient with pairs labelled with matching letters.


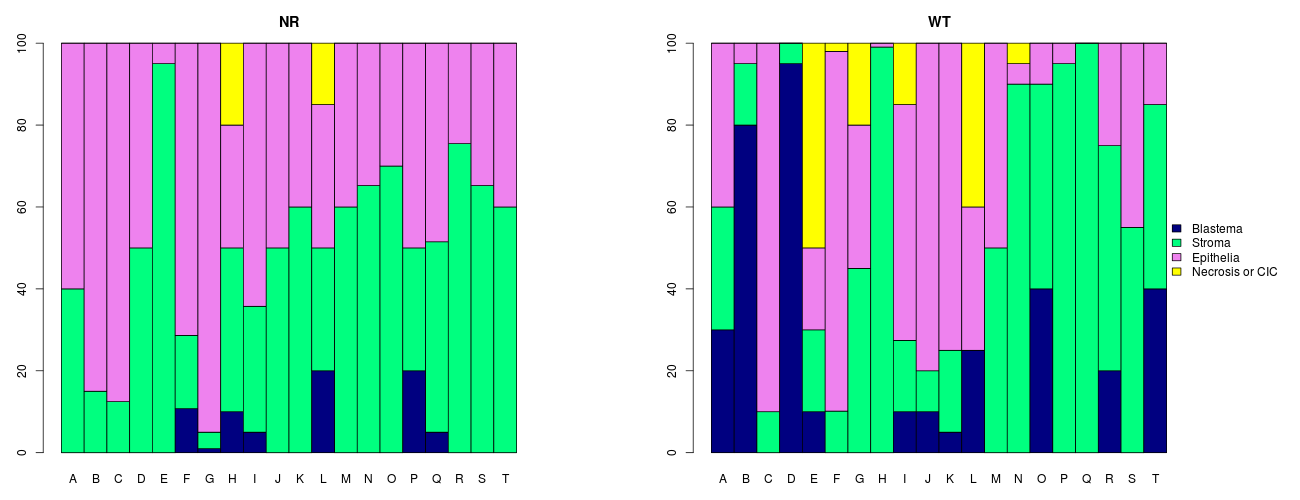


**Table S3: Description of hypermethylated and hypomethylated differentially methylated regions (DMRs) in group-1 Wilms tumour compared to matched nephrogenic rests.**

|  | **Hyper-methylated DMRs** | **Hypo-methylated DMRs** |
| --- | --- | --- |
| Median size (bp) | 527 | 1555.5 |
| Mean size (bp) | 1130.4 | 1749.8 |
| Range (bp) | 108-5,118 | 110-6,780 |
| Total (bp) | 186,507 | 804,907 |
|  |  |  |
| % enrichment: CG island | 0.8 | -22.3 |
| % enrichment: CG shore | 5.5 | -0.5 |
| % enrichment: CG shelf | -2.7 | 2.2 |
| % enrichment: 'other' | -3.6 | 20.6 |
|  |  |  |
| % enrichment: gene body | -3.5 | 14.6 |
| % enrichment: TSS200 | 1.6 | -5.6 |
| % enrichment: TSS1500 | -3.5 | -10.4 |
| % enrichment: 3’ UTR | -2.7 | -1.3 |
| % enrichment: 5’ UTR | -1.6 | -1.6 |
| % enrichment: 1^st^ exon | -1.7 | -3.0 |
| % enrichment: intergenic region | 11.5 | 7.2 |

Genetic features and those relating to CpG islands , shores (within 2kb of islands) and shelves (2-4kb from CpG islands) are extracted from the Illumina 450k annotation. Chromatin data (bivalent, H3K4me3 and H3K27me3 domains) refer to embryonic stem cells (ESCs) [^1^](#_ENREF_1).

**Table S4: Significantly overrepresented biological processes identified by hypomethylated WT-DMRs**

| **Enriched Term in GO biological processes** | **Hypergeometric fold enrichment** | **Raw p-value** |
| --- | --- | --- |
| Axon ensheathment | 2.5 | 1.87E-07 |
| Body morphogenesis | 3.04 | 1.56E-08 |
| Cell chemotaxis | 3.27 | 6.35E-13 |
| Embryonic placenta development | 2.74 | 6.61E-10 |
| Fat cell differentiation | 3.16 | 4.42E-14 |
| Head development | 2.77 | 1.44E-07 |
| Head morphogenesis | 3.54 | 3.44E-10 |
| Intermediate filament-based process | 5.44 | 4.39E-22 |
| Labyrinthine layer development | 4.07 | 9.57E-15 |
| Metanephric nephron development | 3.83 | 2.35E-11 |
| Myelination | 2.57 | 9.16E-08 |
| Myelination in peripheral nervous system | 4.65 | 1.18E-09 |
| Negative regulation of myeloid leukocyte differentiation | 6.43 | 1.48E-22 |
| Nephron development | 2.61 | 1.28E-07 |
| Organ regeneration | 3.86 | 1.14E-13 |
| Palate development | 2.8 | 2.45E-12 |
| Peripheral nervous system development | 2.05 | 6.43E-05 |
| Placenta development | 2.42 | 2.43E-09 |
| Regeneration | 2.39 | 2.63E-08 |
| Regulation of action potential in neuron | 2.19 | 4.73E-06 |
| Regulation of GTP catabolic process | 2 | 5.30E-14 |
| Regulation of myeloid cell differentiation | 2.69 | 3.62E-12 |
| Regulation of myeloid leukocyte differentiation | 3.64 | 2.07E-14 |
| Regulation of purine nucleotide catabolic process | 2.07 | 1.01E-16 |
| Regulation of Ras GTPase activity | 2.14 | 1.27E-10 |
| Regulation of Ras protein signal transduction | 2.02 | 7.60E-14 |
| Schwann cell development | 4.42 | 3.10E-09 |
| Schwann cell differentiation | 3.91 | 3.11E-08 |
| Stem cell development | 2.52 | 2.46E-09 |
| Stem cell differentiation | 2.03 | 2.01E-06 |
| Stem cell maintenance | 2.78 | 8.61E-11 |

**Table S5: Significantly overrepresented biological processes identified by hypermethylated group 2 WT-DMRs**

| **Enriched Term in GO biological processes** | **Hypergeometric fold enrichment** | **Raw p-value** |
| --- | --- | --- |
| Cell adhesion | 2.17 | 3.45E-16 |
| Cell-cell adhesion | 4.83 | 3.00E-44 |
| Homophilic cell adhesion | 12.97 | 5.85E-81 |
| Intracellular receptor mediated signalling pathway | 8.07 | 2.23E-61 |
| Negative regulation of biosyntheic process | 2.35 | 5.46E-30 |
| Negative regulation of cellular biosynthetic process | 2.37 | 1.50E-30 |
| Negative regulation of cellular macromolecule biosynthetic process | 2.51 | 1.22E-33 |
| Negative regulation of cellular metabolic process | 2.13 | 3.76E-26 |
| Negative regulation of gene expression | 2.58 | 1.79E-36 |
| Negative regulation of macromolecule biosynthetic process | 2.44 | 3.08E-32 |
| Negative regulation of macromolecule metabolic process | 2.1 | 2.59E-25 |
| Negative regulation of metabolic process | 2.03 | 6.12E-24 |
| Negative regulation of nitrogen compound metabolic process | 2.5 | 1.70E-33 |
| Negative regulation of nucleobase-containing compound metabolic process | 2.51 | 1.04E-33 |
| Negative regulation of protein metabolic process | 4.19 | 1.92E-31 |
| Negative regulation of RNA metabolic process | 2.61 | 5.53E-36 |
| Negative regulation of transcription from RNA polymerase II promoter | 3.79 | 8.10E-54 |
| Negative regulation of transcription, DNA-dependent | 2.67 | 2.24E-37 |
| Positive regulation of gene expression | 2.06 | 4.97E-22 |
| Positive regulation of RNA metabolic process | 2.06 | 1.76E-21 |
| Positive regulation of transcription from RNA polymerase II promoter | 2.87 | 4.43E-35 |
| Positive regulation of transcription, DNA-dependent | 2.13 | 4.89E-23 |
| Regulation of gene expression, epigenetic | 5.98 | 2.39E-30 |
| Regulation of organelle organisation | 3.57 | 3.43E-34 |
| Regulation of transcription from RNA polymerase II promoter | 2.41 | 9.04E-40 |
| Transcription from RNA polymerase II promoter | 4.87 | 5.31E-49 |

**Table S6: Differentially expressed genes in WT compared to NR**

| **Ensembl ID** | **Gene symbol** | **P-val (adjusted)** | **Average reads NR** | **Average reads WT** |
| --- | --- | --- | --- | --- |
| ENSG00000134917 | ADAMTS8 | 0.01781 | 52.67 | 3.00 |
| ENSG00000091972 | CD200 | 1.03E-09 | 317.67 | 3.00 |
| ENSG00000125734 | GPR108 | 0.01781 | 46.00 | 1.75 |
| ENSG00000110492 | MDK | 0.0008816 | 1.00 | 29.50 |
| ENSG00000162601 | MYSM1 | 0.01284 | 50.67 | 3.75 |
| ENSG00000149294 | NCAM1 | 0.01781 | 6.67 | 57.25 |
| ENSG00000109320 | NFKB1 | 0.02555 | 49.67 | 3.50 |
| ENSG00000138078 | PREPL | 3.68E-06 | 164.67 | 4.50 |
| ENSG00000134198 | TSPAN2 | 0.005864 | 15.67 | 0.00 |

**Table S7: Description of hypermethylated and hypomethylated differentially methylated regions (DMRs) in nephrogenic rests compared to matched normal kidney**

|  | **Hyper-methylated DMRs** | **Hypo-methylated DMRs** |
| --- | --- | --- |
| Median size (bp) | 646 | 1513.5 |
| Mean size (bp) | 1418.5 | 1701.7 |
| Range (bp) | 104-8,088 | 154-5,807 |
| Total (bp) | 487,962 | 483,284 |
|  |  |  |
| % enrichment: CG island | -11.5 | -21.9 |
| % enrichment: CG shore | 9.9 | 2.5 |
| % enrichment: CG shelf | -1.0 | 4.1 |
| % enrichment: 'other' | 2.5 | 15.3 |
|  |  |  |
| % enrichment: ESCs bivalent domain | 10.8 | -1.9 |
| % enrichment: ESCs H3K4me3 only | -3.0 | -15.1 |
| % enrichment: ESCs H3K27me3 only | 3.6 | 1.7 |
| % enrichment: ESCs no H3K27 or H3K4-me3 | -11.4 | 15.3 |
|  |  |  |
| % enrichment: gene body | -1.3 | 16.5 |
| % enrichment: TSS200 | -2.1 | -5.6 |
| % enrichment: TSS1500 | -3.3 | -6.2 |
| % enrichment: 3’ UTR | 1.0 | -0.3 |
| % enrichment: 5’ UTR | 0.0 | -0.8 |
| % enrichment: 1^st^ exon | -2.0 | -2.4 |
| % enrichment: intergenic region | 7.7 | -1.2 |

Genetic features and those relating to CpG islands , shores (within 2kb of islands) and shelves (2-4kb from CpG islands) are extracted from the Illumina 450k annotation. Chromatin data (bivalent, H3K4me3 and H3K27me3 domains) refer to embryonic stem cells (ESCs) [^1^](#_ENREF_1).

**Table S8: Significantly overrepresented biological processes identified by hypomethylated KR-DMRs**

| **Enriched Term in GO biological processes** | **Hypergeometric fold enrichment** | **Raw p-value** |
| --- | --- | --- |
| Actin cytoskeleton organisation | 2.43 | 1.31E-13 |
| Actin filamentbased process | 2.26 | 5.07E-12 |
| Chromosome organisation involved in meiosis | 10.76 | 1.33E-18 |
| Collagen fibril organisation | 6.17 | 3.30E-19 |
| Extracellular matrix organisation | 2.28 | 2.23E-07 |
| Fat cell differentiation | 4.94 | 4.72E-24 |
| Hormone biosynthetic process | 4.43 | 1.72E-13 |
| Hormone metabolic process | 2.52 | 7.31E-08 |
| Induction of apoptosis | 2.24 | 1.70E-13 |
| Induction of programmed cell death | 2.22 | 2.56E-13 |
| Meiosis | 2.75 | 3.61E-07 |
| Meiosis I | 4.43 | 6.91E-11 |
| Meiotic cell cycle | 2.71 | 4.72E-07 |
| Negative regulation of transforming growth factor beta receptor signalling pathway | 6.38 | 2.22E-23 |
| Negative regulation of transmembrane receptor protein serine/threonine kinase signalling pathway | 4.47 | 3.18E-17 |
| Positive regulation of GTPase activity | 2.48 | 2.46E-15 |
| Positive regulation of Ras GTPase activity | 3.31 | 1.89E-16 |
| Regulation of GTP catabolic process | 2.28 | 1.75E-14 |
| Regulation of GTPase activity | 2.25 | 1.39E-13 |
| Regulation of myeloid cell differentiation | 3.14 | 4.19E-12 |
| Regulation of myeloid leukocyte differentiation | 5.43 | 2.51E-21 |
| Regulation of Ras GTPase activity | 2.63 | 5.98E-13 |
| Regulation of Ras protein signal transduction | 2.14 | 1.12E-11 |
| Regulation of transforming growth factor beta receptor signalling pathway | 3.99 | 2.28E-15 |
| Regulation of transmembrane receptor protein serine/threonine kinase signalling pathway | 2.7 | 1.29E-11 |
| Synapsis | 11.04 | 7.12E-19 |
| Synaptonemal complex assembly | 16.25 | 5.36E-23 |
| Synaptonemal complex organisation | 13.37 | 6.78E-21 |
| White fat cell differentiation | 13.41 | 1.05E-42 |

**Table S9: Significantly overrepresented biological processes identified by hypermethylated KR-DMRs**

| \| **Enriched Term in GO biological processes** \| **Hypergeometric fold enrichment** \| **Raw p-value** \| \| --- \| --- \| --- \| \| Amine transport \| 2.99 \| 9.88E-15 \| \| Anterior/posterior axis specification \| 2.5 \| 1.16E-04 \| \| Anterior/posterior axis specification, embryo \| 5.39 \| 4.04E-10 \| \| Anterior/posterior pattern specification \| 2.13 \| 9.87E-11 \| \| Axis specification \| 2.24 \| 2.78E-06 \| \| Blastoderm segmentation \| 4.98 \| 1.36E-10 \| \| Cell-cell adhesion \| 2.08 \| 2.92E-11 \| \| Cell-cell signalling involved in cell fate commitment \| 3.73 \| 1.16E-08 \| \| Developmental induction \| 4.05 \| 2.12E-09 \| \| Embryonic axis specification \| 3.27 \| 1.28E-06 \| \| Embryonic organ morphogenesis \| 2.05 \| 1.18E-10 \| \| Embryonic pattern specification \| 2.17 \| 1.29E-04 \| \| Homophilic cell adhesion \| 4.91 \| 1.09E-29 \| \| Intracellular receptor-mediated signalling pathway \| 3.57 \| 7.02E-25 \| \| Negative regulation of kinase activity \| 3.39 \| 7.73E-23 \| \| Negative regulation of organelle organisation \| 2.94 \| 1.40E-15 \| \| Negative regulation of protein kinase activity \| 3.75 \| 9.52E-26 \| \| Negative regulation of protein modification process \| 2.52 \| 3.46E-11 \| \| Negative regulation of transferase activity \| 3.35 \| 6.67E-24 \| \| Nitrogen compound transport \| 3.22 \| 2.34E-19 \| \| Peptidyl-proline modification \| 4.17 \| 5.81E-10 \| \| Protein peptidyl-prolyl isomerisation \| 4.6 \| 6.36E-11 \| \| Regulation of adaptive immune response based on somatic recombination of immune receptors built from immunoglobulin superfamily domains \| 2.02 \| 3.71E-03 \| \| Regulation of T-helper 1 type immune response \| 7.62 \| 2.07E-11 \| \| Transition metal ion transport \| 3.55 \| 9.90E-10 \| |  |  |
| --- | --- | --- | --- | --- | --- | --- | --- | --- | --- | --- | --- | --- | --- | --- | --- | --- | --- | --- | --- | --- | --- | --- | --- | --- | --- | --- | --- | --- | --- | --- | --- | --- | --- | --- | --- | --- | --- | --- | --- | --- | --- | --- | --- | --- | --- | --- | --- | --- | --- | --- | --- | --- | --- | --- | --- | --- | --- | --- | --- | --- | --- | --- | --- | --- | --- | --- | --- | --- | --- | --- | --- | --- | --- | --- | --- | --- | --- | --- | --- | --- |

**References**

**1 Pan, G. *et al.* Whole-Genome Analysis of Histone H3 Lysine 4 and Lysine 27 Methylation in Human Embryonic Stem Cells. *Cell Stem Cell* 1, 299-312, doi:**[**http://dx.doi.org/10.1016/j.stem.2007.08.003**](http://dx.doi.org/10.1016/j.stem.2007.08.003) **(2007).**
